# Etude épidémio-clinique des diarrhées aiguës à rotavirus chez les nourrissons à l'hôpital Jason Sendwe de Lubumbashi, République Démocratique du Congo

**DOI:** 10.11604/pamj.2015.21.113.5737

**Published:** 2015-06-10

**Authors:** Maguy Kabuya Sangaji, Olivier Mukuku, Augustin Mulangu Mutombo, Paul Makan Mawaw, Edouard Kawawa Swana, Benjamin Kasongo Kabulo, André Kabamba Mutombo, Stanis Okitotsho Wembonyama, Oscar Numbi Luboya

**Affiliations:** 1 Département de Pédiatrie, Faculté de Médecine, Université de Lubumbashi, République Démocratique du Congo; 2 Département de Santé Publique, Faculté de Médecine, Université de Lubumbashi, République Démocratique du Congo

**Keywords:** Rotavirus, diarrhée aiguë, nourrisson, déshydratation, Lubumbashi, RDC, Rotavirus, acute diarrhea, infant, dehydration, Lubumbashi, DRC

## Abstract

**Introduction:**

Le rotavirus est un problème de santé publique, non seulement dans les pays en développement où tous les enfants sont infectés avant l’âge de deux à trois ans mais aussi dans les pays développés où les conditions d'hygiène sont bonnes. La présente étude est la première à fournir des informations sur la prévalence de l'infection à rotavirus dans les diarrhées aiguës des nourrissons dans la ville de Lubumbashi. Elle s'est fixée comme objectifs de déterminer la fréquence hospitalière ainsi que la saisonnalité, les caractéristiques sociodémographiques, cliniques et évolutives de l'infection à Rotavirus chez les nourrissons admis à l'hôpital Jason Sendwe de Lubumbashi pour une diarrhée aiguë.

**Méthodes:**

Il s'agit d'une étude descriptive et transversale menée pendant la période allant du 1^er^ janvier au 31 décembre 2012. Les paramètres épidémio-cliniques et évolutifs (âge, sexe, saison, signes cliniques, nombre journalier de selles et évolution) des enfants diagnostiqués positifs au rotavirus ont été comparés à ceux des enfants dont le test au rotavirus était négatif. Le degré de signification était de 5%.

**Résultats:**

Nous avons récolté 193 cas de diarrhées aiguës dont 104 nourrissons étaient infectés par le rotavirus soit 53,8%. Des taux élevés des diarrhées à rotavirus sont enregistrés au cours de la saison sèche comparativement à la saison des pluies (p0,05). Par ailleurs, les enfants infectés par le rotavirus étaient 6 fois plus susceptibles de présenter une déshydratation modérée/sévère (p0,05).

**Conclusion:**

Le rotavirus est confirmé dans la ville de Lubumbashi et touche souvent les enfants d’âge ≤12 mois, pendant la saison sèche sans distinction de sexe et conduit rapidement à une déshydratation modérée/sévère. Une prise en charge adaptée et précoce permet d’éviter les décès et l'assainissement du milieu, le lavage des mains, la prise d'eau potable et la vaccination contre le rotavirus sont les mesures préventives les plus efficaces contre les rotavirus et à conseiller dans notre communauté.

## Introduction

En 2013, l'Organisation Mondiale de la Santé définissait la diarrhée aiguë comme l’émission d'au moins trois selles molles ou liquides par jour (ou l’émission trop fréquente que ce qui est habituel pour le sujet atteint) [1]. Dans les pays industrialisés, les diarrhées infectieuses restent la deuxième cause d'admission en milieu hospitalier et la cause la plus fréquente de consultation en pédiatrie [2]. En France, les diarrhées aiguées représentent une morbidité importante à l'origine de 2,9 à 3 millions de consultations annuelles chez le généraliste [3]. La diarrhée aiguë de l'enfant est un problème de santé publique à l’échelle mondiale et est responsable d'une mortalité considérable dans les pays en voie de développement. Les enfants de moins de 5 ans présentent 1,3 et 2,3 épisodes annuelles de diarrhée aiguë [1]. Parmi les principales causes infectieuses de la diarrhée aiguë, les virus dont le rotavirus occupent une place importante. Les gastro-entérites virales sont à l'origine de plus de la moitié de diarrhée infectieuse aux Etas Unis comme en Europe [4]. Le rotavirus est le principal agent causal des formes graves de la diarrhée aiguë chez l'enfant de moins de 2 ans [5]. Le rotavirus est un problème de santé publique, non seulement dans les pays en développement où tous les enfants sont infectés avant l’âge de deux à trois ans mais aussi dans les pays développés où les conditions d'hygiène sont bonnes. Mais dans les pays en développement, le problème reste important à cause de certains facteurs comme le dysfonctionnement des systèmes sanitaires, la malnutrition importante des enfants, les budgets faibles alloués à la santé, l'insuffisance en équipements et en personnel hautement qualifié pour prendre correctement en charge les enfants malades [1]. En 2003, le fonds des nations unies pour l'enfance (UNICEF) déclare qu'en RDCongo la diarrhée aiguë est responsable d'au moins 13,5% de mortalité infantile et en 2010, le rapport du « Multi Indicators Cluster Survey » (MICS-RDC) relevait que la prévalence de la diarrhée était de 15% dans la province du Katanga et de 18% au niveau national [6]. En effet plusieurs études menées ailleurs démontrent clairement la responsabilité de ce virus [7–9]. En RDCongo, selon Nganda, en 1986 le rotavirus était responsable de 25% de mortalité à Kinshasa par déshydratation [10].

La présente étude est la première à fournir des informations sur la prévalence de l'infection à rotavirus dans les diarrhées aiguës des nourrissons dans la ville de Lubumbashi. Elle s'est fixé comme objectifs de déterminer la fréquence en milieu hospitalier ainsi que la saisonnalité, les caractéristiques sociodémographiques, cliniques et évolutives de l'infection à Rotavirus chez les nourrissons âgés de 2 ans ou moins vivant dans la ville de Lubumbashi admis à l′hôpital Jason Sendwe pour une diarrhée aiguë.

## Méthodes

Il s'agit d'une étude descriptive et transversale menée au cours de la période allant du 1^er^ janvier au 31 décembre 2012 à l'hôpital général Jason Sendwe de Lubumbashi en République Démocratique du Congo. Il s'agit d'un échantillonnage exhaustif de tous les nourrissons de 2 ans ou moins admis pour diarrhée aiguë et récoltés de façon consécutive. Notre échantillon est constitué de 193 cas de diarrhée aiguë dont 104 infectés par le rotavirus. Une fiche a été élaborée pour la collecte des données et comprenait un questionnaire qui a aidé à obtenir les informations recherchées. Les informations ont été recueillies cas par cas en consultant le registre des soins et les résultats de laboratoire.

La recherche virologique du rotavirus a été effectuée dans les selles par la méthode d'ELISA (enzyme-linked immunosorbent assay). Elle a été réalisée au laboratoire de l'hôpital Jason Sendwe. Tous les résultats positifs ont été envoyés respectivement au grand laboratoire de Lubumbashi qui est le laboratoire provincial de référence pour la confirmation. Ainsi la positivité au rotavirus a été confirmée dans les 2 laboratoires (ELISA positif au rotavirus dans les selles) pour chacun des malades. Si un laboratoire déclarait un cas négatif, ce dernier était automatiquement écarté de notre série.

Les critères d'inclusion ont été les suivants: enfant âgé de 2 ans ou moins; présentant une diarrhée aiguë (durée de la diarrhée inférieure à 7 jours); avoir réalisé un examen virologique des selles. Les critères d'exclusion sont ci-après: avoir l’âge supérieur à 2 ans; durée de la diarrhée égale ou supérieure à 7 jours; n'avoir pas effectué un examen virologique à rotavirus.

Les paramètres étudiés sont: l’âge, le sexe, le mois, la saison, les signes cliniques, le nombre de selles par jour, les résultats de laboratoire de selles et l'issue (évolution). Les données ont été saisies et analysées grâce aux logiciels Microsoft Excel 10 et Epi-Info 2011. Les paramètres des enfants diagnostiqués positifs au rotavirus ont été comparés à ceux des enfants dont le test au rotavirus était négatif. L'analyse a utilisé la moyenne et l’écart type, la médiane, le pourcentage, le test de Khi2, le test de Student, l'odds ratio et les intervalles de confiance à 95%. Le degré de signification était de 5%.

*Considérations éthiques:* nous avons demandé et obtenu le consentement libre et éclairé de toutes les femmes qui accompagnaient les enfants et nous avons gardé la confidentialité des résultats et des renseignements reçus. La recherche pour réaliser cette étude a été autorisée par le comité d’éthique de l'Université de Lubumbashi.

## Résultats

**Figure 1:**
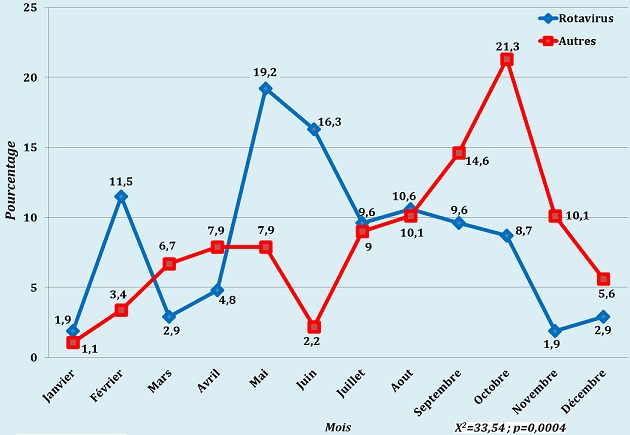
Répartition des cas de diarrhées selon le mois d'admission et le germe en cause X^2^=33,54; p = 0,0004

Sur un total de 193 enfants admis pour diarrhées aigües, 104 l'ont été pour diarrhée à Rotavirus, ce qui donne une proportion de 53,9%. De la Figure 1, il ressort que la survenue de diarrhées à rotavirus est liée au mois de l'année et l'analyse statistique montre une association significative (X^2^=33,54; p = 0,0004). Le Tableau 1 donne les différentes répartitions des patients en ce qui concerne le sexe, l’âge, la saisonnalité, les signes cliniques ainsi que l’évolution.

**Tableau 1:** Répartition des aspects épidémio-cliniques et évolutifs des nourrissons présentant la diarrhée aiguë selon le germe en cause

Paramètre	Rotavirus (n = 104)		Non rotavirus (n = 89)		Total (n = 193)		p	OR [ICà95%]
	n	%	n	%	n	%		
Sexe								
Féminin	54	51,9	47	52,8	101	52,3	-	1
Masculin	50	48,1	42	47,2	92	47,7	0,9826	1,03 [0,6-1,8]
Age								
≤3 mois	19	18,4	12	13,5	31	16,1	0,0166	4,0 [1,4-11,6]
4-6 mois	40	38,5	23	25,8	63	32,6	0,0023	4,4 [1,8-11,2]
7-12 mois	36	34,6	31	34,8	67	34,7	0,0294	2,9 [1,2-7,4]
>12 mois	9	8,6	23	25,8	32	16,6	-	1
Moyenne (mois)	6,9±4,5		9,6±5,9				0,0006	
Saison								
Sèche	73	70,2	46	51,7	119	61,7	0,0128	2,2 [1,2-3,9]
Pluvieuse	31	29,8	43	48,3	74	38,3	-	1
Nombre journalier de selles							
<6	42	40,4	52	58,4	94	48,7	-	1
≥6	62	59,6	37	41,6	99	51,3	0,0185	2,07 [1,16-3,68]
Moyenne	6,2±2,5		5,2±2,3				0,0134	
Fièvre								
Oui	86	82,7	71	79,8	157	81,3	0,7113	1,21 [0,58-2,50]
Non	18	17,3	18	20,2	36	18,7	-	1
Vomissements								
Oui	88	84,6	71	79,8	159	82,4	0,4899	1,39 [0,66-2,92]
Non	16	15,4	18	20,2	34	17,6	-	1
Léthargie								
Oui	34	32,7	27	30,3	61	31,6	0,8449	1,11 [0,60-2,05]
Non	70	67,3	62	69,7	132	68,4	-	1
Degré de déshydratation							
Sévère/modérée	102	98,1	79	88,7	181	93,8	0,0134	6,45 [1,37-30,30]
Légère	2	1,9	10	11,3	12	6,2	-	1
Admission en urgence							
Oui	103	99,0	88	98,9	191	99,0	1,0000	0,85 [0,07-18,98]
Non	1	1,0	1	1,1	2	1,0	-	1
Hospitalisation								
Oui	103	99,0	81	91,0	184	95,3	0,0126	10,17 [1,24-83,0]
Non	1	1,0	8	9,0	9	4,7	-	1
Issue								
Guérison	103	99,0	89	100,0	192	99,5	-	
Décès	1	1,0	0	0,0	1	0,5	1,000	undefined

Le sexe féminin est légèrement prédominant avec 51,9% des cas et le sexe ratio F/M est de 1,08 chez les enfants infectés par le rotavirus et il en est de même chez les enfants infectés par d'autres virus (52,8%) mais avec un sexe ratio F/M 1,12. La comparaison de ces deux proportions ne donne pas de différence statistiquement significative (p = 0,9826).

L’âge moyen est de 6,9±4,5 mois et s’étale entre 1 et 24 mois (Q1 = 4 mois et Q2 = 9 mois) chez les enfants infectées par le rotavirus alors qu'il est de 9,6±5,9 mois allant de 2 à 24 mois (Q1 = 5 mois et Q2 = 13 mois) chez ceux dont la diarrhée était due aux autres virus. En comparant ces moyennes d’âge, le test de Student montre une différence statistiquement significative (t = 3,51; p = 0,0006). Près de 92% de nos patients infectés par le rotavirus avaient un âge inférieur ou égal à 12 mois contre 74% chez ceux infectés par d'autres virus. Comme nous le montre le Tableau 1, nous remarquons que les enfants âgés de plus de 12 mois sont les moins infectés par le rotavirus et lorsque nous comparons les autres tranches d’âge par rapport à celle-ci, nous notons une différence statistiquement significative (p

Septante virgule deux pourcent des cas de diarrhées à rotavirus sont survenus pendant la saison sèche contre 51,7% des cas de diarrhées dues aux autres germes; la comparaison de ces deux proportions est statistiquement significative (p = 0,0128).

Le nombre journalier de selles diarrhéiques est de 6,2±2,5 chez les enfants infectés du rotavirus alors qu'il est de 5,2±2,3 chez les enfants infectés des autres micro-organismes. La comparaison de ces deux moyennes montre une différence statistiquement significative (t = 2,49; p = 0,0046). La proportion des enfants dont le nombre journalier de selles est ≥6 est de 41,6% dans le groupe des autres micro-organismes contre 59,6% dans le groupe de rotavirus, ces derniers présentant un risque de 2 fois d'aller à selles plus de 6 fois par jour (OR = 2,07; ICà95%: 1,16-3,68; p = 0,0134). Les diarrhées à rotavirus conduisent plus à une déshydratation sévère/modérée que les diarrhées dues aux autres virus et ceci de manière statistiquement significative (OR = 6,45; ICà95%: 1,37-30,30; p = 0,0134).

En ce qui concerne les signes d'accompagnement de la diarrhée, la comparaison entre les nourrissons infectés et ceux non infectés par le rotavirus montre des proportions élevées des vomissements, de la fièvre et de la léthargie chez les infectés mais la différence n’était pas statistiquement significative (p > 0,05).

De notre série, il ressort que l'admission en urgence n’était pas différente statistiquement entre les enfants infectés au rotavirus et ceux infectés par d'autres germes; mais quant à ce qui concerne l'hospitalisation, nous constatons qu'il existe une différence statistiquement significative entre ces deux groupes d'enfants et que ceux qui sont infectés par le rotavirus ont un risque de 10,71 fois d’être hospitalisés que ceux infectés par d'autres germes (OR = 10,17; IC95%: 1,24-83,0).

S'agissant de l’évolution de nos patients, nous avons enregistré un décès (1% des cas) chez les enfants infectés par le rotavirus et aucun décès chez ceux non infectés par le rotavirus (p = 1,000).

## Discussion

Au cours de la période étudiée, nous avons enregistrés 193 enfants admis pour diarrhées aigües, 104 ont été testé positif au Rotavirus. Les résultats ont confirmé que le rotavirus est une cause majeure d′hospitalisation pour gastro-entérite aiguë chez les nourrissons, ce qui représente **53,9%** des cas. L′incidence du rotavirus est presque similaire dans les pays développés et en développement et varie d'un pays à un autre voire même dans un pays d'une région à une autre [11]. De nombreuses études ont montré le rôle important des rotavirus comme une cause de diarrhée chez les enfants dans les pays développés comme dans les pays en développement dont la plupart des cas se sont produits chez les enfants de moins de 5 ans [12–14]. Notre prévalence est proche à celles trouvées dans des études récentes menées dans certains pays africains [15, 16]. Dans deux régions du Nord du Cameroun, une étude réalisée par Ndze rapporte une prévalence de 42,8%, taux relativement supérieur à 21,9% rapporté dans la région ouest du même pays [17]. Mais des fréquences plus basses ont été aussi rapportées sur le continent africain; c'est le cas d'une étude menée au Nigeria par Junaid qui enregistre 13,8% [18]. De 2007 à 2008, la surveillance constante de la diarrhée à rotavirus à Shiraz (Iran), a montré une prévalence de 34,8% chez les enfants de moins de 5 ans souffrant de gastroentérite [19]. En Europe occidentale, entre 2004 et 2005, environ 10,4% à 36,0% des enfants âgés de moins de cinq ans ont été admis une gastro-entérite aiguë due au rotavirus [20]. Dans une étude prospective menée dans cinq pays de l′Union Européenne (France, Allemagne, Italie, Espagne et Royaume-Uni) chez les enfants âgés de moins de 5 ans, le rotavirus représentait 56,2% de toutes les causes d'hospitalisation de cas de gastro-entérite aiguë, allant de 33,2% en Italie à 64,4% en France [21]. En Europe centrale et orientale, parmi la population pédiatrique, l′infection à rotavirus représente entre 22% et 55% des cas de gastro-entérite aiguë [22–24]. Certaines différences entre les prévalences enregistrées pourraient s'expliquer par la technique de laboratoire utilisée pour détecter le rotavirus car certains utilisent le RT-PCR (reserve transcription-polymerase chain reaction) [17] et d'autres, comme nous, recourent à la méthode par dosage immuno-enzymatique (test ELISA) [18].

Bien que nos résultats montrent la présence de l'infection à rotavirus tout le long de l'année, les taux élevés sont enregistrés au cours de la saison sèche comparativement à la saison des pluies qui enregistre les taux bas et nous avons noté une différence statistiquement significative (p17,25-28]. Ce constat est identique à celui notifié en Asie du Sud dans une méta-analyse faite par Jagai sur la saisonnalité de l'infection à rotavirus trouve les taux plus élevés pendant les mois les plus froids et secs [29]. Notre étude a démontré qu′il y avait une corrélation significative entre la répartition saisonnière et l'infection à rotavirus. La gastroentérite à rotavirus s′est produite tout au long de l′année, avec plus de cas survenant en saison sèche dans notre milieu correspondant à la période froide. Dans les régions sous climats tempérés, des nombreuses études ont montré que les infections à rotavirus surviennent surtout au cours de l'hiver correspondant à la saison froide; c'est le cas de l′Iran [30], la Chine [31], l'Europe [32–34] et les Etats-Unis [11].

Près de 92% de nos patients infectés par le rotavirus ont un âge inférieur ou égal à 12 mois avec un pic entre 4 et 12 mois et l’âge moyen est de 6,9 ± 4,5 mois. Bonkoungou, lui trouve 94,2% des cas chez les enfants de moins de 2 ans avec un âge moyen de 8 mois [26]. L’étude menée au Cameroun par Mbuh rapporte 40% des cas entre 7 et 9 mois d’âge [35]. En Ouganda, Nakawesi note une moyenne d’âge de 10 mois [15]. Au Nigeria, Jumaid trouve un pic d'infection à Rotavirus chez les enfants âgés de 7 à 12 mois [18]. Comme nous le voyons, l'infection à rotavirus touche principalement les enfants âgés de moins de 2 ans avec un pic d'incidence chez les enfants de 6-11 mois d′âge. Ceci pourrait être expliqué par l′effet protecteur des anticorps maternels chez les moins de 6 mois ainsi que le développement de l′immunité naturelle après des infections répétées chez les enfants de plus de 2 ans [36, 37]. Etant donné que le taux d′allaitement maternel commence à diminuer au bout de 6 mois, Clemens souligne que les effets protecteurs de l′allaitement maternel semblent aussi diminuer avec l′âge [38].

L’étude montre que l'infection à rotavirus était plus retrouvée chez les nourrissons de sexe féminin que chez leurs homologues masculins mais sans aucune différence statistique entre les deux sexes. Ce résultat concorde avec ceux trouvé par Ndze au Cameroun [17], Bonkoungou au Burkina-Faso [26], Saravanan en Inde [39] et Temu en Tanzanie [40] mais contraste avec les observations faites par d′autres études dans lesquelles une prépondérance statistiquement significative de l′infection à rotavirus a été observée chez les enfants de sexe masculin allant de 60% à 70% [19, 41, 42]. Selon Fischer, la prédilection du sexe masculin pourrait être expliquée par des facteurs génétiques et immunologiques; les hommes sont plus susceptibles à développer une forme grave de diarrhée nécessitant une hospitalisation bien que les deux sexes soient infectés à la même vitesse [43]. Si cette différence est due à la susceptibilité de sexe ou par hasard, elle reste cependant discutable et doit être approfondie.

L′infection à rotavirus chez les nourrissons et les jeunes enfants peut conduire à une diarrhée grave, de déshydratation, un déséquilibre électrolytique et une acidose métabolique [11]. La comparaison entre les nourrissons infectés et les non infectés par le rotavirus montre des proportions élevées des vomissements, de la fièvre et de la léthargie chez les infectés bien que la différence n’était pas statistiquement significative. Par ailleurs, les enfants infectés par le rotavirus étaient 6 fois plus susceptibles de présenter une déshydratation modérée/sévère (p

Cette étude a des faiblesses ci-après: elle n'a pas été menée dans la communauté mais uniquement à l'hôpital Sendwe. A cause des ressources limitées, l’étude n'a pas concerné les autres structures des soins de la ville de Lubumbashi. De même cette recherche n'a pas permis de diagnostiquer les différentes souches du rotavirus qui existeraient à Lubumbashi, ce qui constitue une partie de notre perspective de recherche pour l'avenir. Cependant la force de notre étude est certaine. Il s'agit de la première enquête menée à Lubumbashi et dans la province sur la diarrhée aiguë qui démontre à suffisance le rôle du rotavirus dans notre milieu. Les résultats de cette étude serviront à court ou à moyen terme aux autorités sanitaires dans leur prise de décision cohérente sur le contrôle adéquat de la diarrhée aiguë à rotavirus à Lubumbashi, au Katanga et au pays surtout dans le volet préventif à travers un vaccin polyvalent efficace et efficient et aux autres chercheurs.

## Conclusion

Le rotavirus est bel et bien présent dans la ville de Lubumbashi et touche le plus souvent les enfants d’âge ≤12 mois, pendant la saison sèche sans distinction de sexe et conduit rapidement à une déshydratation modérée/sévère. Une prise en charge adaptée et précoce permet d’éviter les décès et l'assainissement du milieu, le lavage des mains, la prise d'eau potable et la vaccination contre le rotavirus sont les mesures préventives les plus efficaces contre les rotavirus et à conseiller dans notre communauté.
